# Neurocognitive Lag in School-Aged Children Living With HIV in India and Its Relevance

**DOI:** 10.7759/cureus.16110

**Published:** 2021-07-02

**Authors:** Vishwanath ., Alok Hemal, Manju Nimesh, Meetu Singh, Sheetal Agarwal

**Affiliations:** 1 Pediatrics, Atal Bihari Vajpayee Institute of Medical Sciences and Dr. Ram Manohar Lohia Hospital, New Delhi, IND

**Keywords:** children living with hiv, neurocognitive deficit, cd4 count, raven score, standard progressive matrices

## Abstract

Objective

Objective assessment of neurocognitive lags in pediatric HIV patients and its correlation with various clinical, social and familial factors.

Methods

Ninety-eight school-aged children living with HIV (CLHIV) (age 7-18 years) attending regional pediatric HIV clinic were observed for neurocognitive lag using Raven’s Standard Progressive Matrices by the same trained instructor. Sociodemographic data, mode of transmission, clinical staging, CD4 count, highly active antiretroviral therapy (HAART) duration were recorded and analyzed in the well-performing group and under-performing group.

Results

29.6% of children had definitive neurocognitive lag. The proportion of older children (11-18 years) in the under-performing group was significantly high (P = 0.007). The mean CD4 counts were low in the under-performing group (P = 0.001). Other socioeconomic factors could not be specifically correlated with neurocognitive lag in either of the groups.

Conclusion

CLHIV has a significant neurocognitive lag, which is accentuated in the upper age group. Findings point toward declining intellectual gains with increasing age in CLHIV.

## Introduction

Neurocognitive manifestations are more frequent and more intense in pediatric HIV disease as compared to their adult variants. Early CNS seeding of virus and progressive neuronal injury is responsible for such severe neurodevelopmental outcomes in children. Overall survival of such children has prolonged with advancements in diagnostic and medical treatment of HIV infection. Neurocognitive lags in situations of improved survivals negatively affect scholastic performance and social integration [1,2].

The influence of factors affecting neurocognitive development in children living with HIV (CLHIV) is likely to be different in the Indian context as against western countries due to different sociocultural factors and policy-related issues, and actual outcomes may be different. Literature is scarce in evaluating neurocognitive function among school-aged CLHIV in the Indian population [3].

We objectively assessed the neurocognitive function of school-aged CLHIV along with its correlation with various factors. Such an assessment may guide policy planning towards specific aspects like follow-up assessments, selection of schooling techniques, and social support modalities.

## Materials and methods

CLHIV attending regional pediatric HIV clinic of tertiary level teaching hospital from November 2017 to April 2019 were prospectively observed. The pediatric HIV clinic was annexed to the National AIDS Control Program (NACP) and followed all national guidelines [4]. The study was approved by the institutional ethical review board and written informed consent was obtained from all parents or caregivers for enrolment in the study.

Patients with a confirmed diagnosis of HIV infection in the age group of 7-18 years were included in the study. All children were managed as per the National Guidelines for Paediatric HIV/AIDS care [4]. Children with pre-existing clinical entities which could have a direct impact on neurocognitive function like a perinatal neurological insult, prematurity, congenital brain anomalies, traumatic head injury, active intracranial conditions, like tuberculosis, tumors, neuropsychiatric illness present, chromosomal anomalies, seizures, etc., were excluded from the study using meticulous clinical and laboratory investigations.

Modes of transmission, clinical staging, CD4 counts, highly active antiretroviral therapy (HAART) details were documented for each patient. Disease staging was done as per the WHO criteria [5]. Sociodemographic data of the patients and caregivers were recorded. The socioeconomic status of the family was assessed using a Modified Kuppuswamy scale [6].

A detailed neurological examination was done for each patient. The neurocognitive assessment was done using Raven’s Standard Progressive Matrices (SPM). Colored progressive matrices were used for children up to the age of 7-10, while classic SPM were used for children >11 years of age [7]. The children and caregivers were informed and explained the test to be commenced. All assessments were done by the same trained instructor in a comfortable ambience.

Raw scores of all the patients were checked independently by two independent observers. Colored progressive matrices scores were converted to SPM equivalent for analysis purposes based on Raven’s conversion tables [7]. Each child’s raw score was converted to percentile score and graded in five grades of neurocognitive capabilities based on age-matched normative data of Indian children [8].

Children with Raven’s Grades 4 & 5 (scores ≤25th percentile) were counted as having significant neurocognitive lag [7,8]. Children were divided into two cohorts of the under-performing group (Raven’s Grades 4 & 5) and the well-performing group (Raven’s Grades 1-3) for analysis purposes. Additionally, caregivers were interviewed about the scholastic performance of children using a semi-structured questionnaire.

Statistics analysis was done using the SPSS version 20.0 software (IBM Corporation, Armonk, NY, USA). Continuous variables were presented as mean and categorical variables expressed as frequencies and percentages. Continuous variables were analyzed using the student’s t-test. The Pearson's chi-square test or Fisher’s exact test were used to determining the relationship between categorical variables. Statistical significance was set at a probability level of 0.05.

## Results

Ninety-eight children (male 67, female 31) with a mean age of 12.3 years (range; 7-18 years) were enrolled in the study. Either both or one biological parent was the primary caretaker in 88.8% of children, while six (6.1%) and five (5.1%) of children were being cared for by their proximal family and non-family support agencies, respectively, after the demise of their biological parents. Ninety (90.8%) of children acquired the infection through the vertical route of transmission. All patients were from urban and semi-urban localities. Clinically, 94 (94.9%) of children belonged to WHO stage 1. The mean HAART duration was 6.9 years (range; 1 month-14 years) (Table 1).

**Table 1:** Sociodemographic and clinical characteristics of patients ART - antiretroviral therapy

Parameter		Well-performed Group	Under-performed Group	Total	Significance level (P-value )
No of patients		69 (70.4%)	29 (29.6%)	98 (100%)	
Age (mean, in years )		11.7	13.6	12.3	
Age distribution	7-10 years	35	6	41 (41.8%)	0.007
	11-18 years	34	23	57 (58.2%)	
Gender	Male	45	22	67 (68.4%)	0.301
	Female	24	7	31 (31.6%)	
Caretaker	Parent - either one or both	62	25	87 (88.8%)	0.848
	Proximal family	4	2	6 (6.1%)	
	Non-family agency	3	2	5 (5.1%)	
Parent deceased status	Both parents alive	44	17	61 (62.2%)	0.704
	One parent alive	20	11	31 (31.6%)	
	Both parents deceased	5	1	6 (6.1%)	
Socioeconomic status	Lower	37	21	58 (59.2%)	0.108
	Upper lower	8	4	12 (12.2%)	
	Middle	24	4	28 (28.6%)	
Education status	Uneducated/not enrolled	1	1	2 (2.1%)	0.061
	Primary	41	8	49 (50.0%)	
	Secondary	21	19	40 (40.8%)	
	Senior secondary or higher	6	1	7 (7.1%)	
Route of transmission	Vertical	62	27	89 (90.8%)	0.403
	Parenteral	2	0	3 (2.0%)	
	Other modes	3	0	2 (2.0%)	
	Unknown	2	2	4 (4.1%)	
WHO staging	1	66	27	93 (94.9%)	0.805
	2	1	1	2 (2.0%)	
	3	2	1	3 (3.1%)	
ART duration (in years)		6.5	7.9	6.9	0.077
CD4 count (per mm^3^)		846.2	611.1	776.6	0.001
CD4 count distribution (per mm^3^)	Less than 200	3	3	6 (6.1%)	0.001
	200-349	4	1	5 (5.1%)	
	350-499	3	9	12 (12.2%)	
	≥500	59	16	75 (76.5%)	

29.6% of children had definitive neurocognitive lag with Raven scores below 25th percentile (Grades 4 & 5), while an additional 31.6% of children had scores below 50th percentile (Grade 3−). Neurocognitive lag was significantly higher (40.3%; n=23) in the 11-18 years age group as compared to the 7-10 years age group (P = 0.007) (Table 2).

**Table 2:** Raven’s Standard Progressive Metrics scores

Raven Grade	7-10 years	11-18 years	Total	P-value
Grade 1	5	0	5	0.007
Grade 2	2	4	6	0.664
Grade 3+	15	12	27	0.090
Grade 3-	13	18	31	0.989
Grade 4	3	18	21	0.004
Grade 5	3	5	8	0.795

97.7% of children were enrolled in formal school education programs, while the remaining 2.2% of children were not attending schools either as school dropouts or never started schooling. 76.5% of parents and caregivers perceived their wards as problem learners and need additional scholastic efforts to enhance learning. Various reasons enlisted by parents and caregivers for such poor scholastic performance were lack of stimulating social and home environment, absenteeism from school for medical ailments, poor understanding, social stigma, nutritional status, etc.

The mean CD4 count in the Under-performing group was 611.1 cells/mm^3^ in comparison to the mean value of 846.2 cells/mm^3^ in the well-performing group, which was estimated to be statistically significant (P = 0.001). Parental survival status, type of caregiver, route of transmission, WHO clinical grade, HAART duration, and socioeconomic status could not be significantly correlated with neurocognitive lag in either of the groups probably due to smaller sample size.

## Discussion

Poor neurocognitive functioning was noted among the HIV-infected children in our study. We observed a higher prevalence of neurocognitive lag in the older age group (11-18 years) in our study group. Accentuated neurocognitive lag in higher age group pointed to declining intellectual gains with increasing age and declining influence of various factors building general intelligence of a growing child.

HIV permeates the blood-brain barrier early during primary systemic viremia and results in irreversible neuronal injury to the developing brain. Such an early seeding of brain macrophages and microglia continues to cause progressive neuronal damage along with an additional contribution from virus-induced immune responses [9,10].

This pattern of early neurocognitive impairment even before immunosuppression was corroborated in our study with the presence of intellectual impairment at a young age much before significant immunosuppression. The majority of younger patients in our study were in WHO clinical stage 1 without any evidence of immunosuppression but accounted for 20.7% of cases with neurocognitive lag.

Early ART along with good medical support partially mitigate the neurocognitive impairment in HIV-infected children, but some form of pre-existing deficits persist. Such deficits tend to be compounded by multiple socioeconomic factors over the developing years and explain a higher prevalence of neurocognitive in the older age group [11]. Patients with low CD4 count were found to have higher neurocognitive manifestations pointing toward higher viral activity and immunosuppression effects in brain parenchyma. Low CD4 counts also point toward the quality and compliance of HAART [12].

It has been found that apart from direct viral neural toxicity and recurrent opportunistic infections multiple factors could influence the neurocognitive outcome of the developing brain. These can be socioeconomic status, family structure, literacy status, child-rearing practices of community, child-caregivers relationship, social and family support, stigma, life stresses, the physical and mental health of family members, availability of resources, and national policies for HIV patients [13,14]. The literature identifies these as important interlinked determinants, but our study could not find a significant association probably due to the smaller sample size and lack of prospective observation.

Comparison of our pediatric HIV care program with that of developed nations offers some insights. National HIV/AIDS Strategy of USA incorporates family-centered care of pediatric patients, along with identification of schooling methods, family support in livelihood earnings, and periodic measurement of health outcomes. The national program also recruits care providers from varieties of sub-specialties. Sub-specialty care providers are entrusted with the task of periodic neurocognitive development of each CLHIV and appropriate recommendation for medical and non-medical aspects of child development. It also prescribes for enrollment of children in schools based on scholastic performance [15].

The National AIDS control program in India seems to have a less affirmative stance towards monitoring of neurocognitive development of HIV-infected children and rehabilitative measures for neurocognitively impaired children. It has prudently evolved from CD4 count-based HAART to the early institution of HAART to arrest the development of various immune response-related ailments. But the integration of family-based care, community support, and economic upliftment of families in the national program is still in the nascent phase [16].

The present study presents a dataset based on a cross-sectional assessment of CLHIV in the school-age group. Longitudinal assessment of the neurocognitive function and development pattern in CLHIV with a larger sample size may reveal the exact dynamics and impact of various socioeconomic factors.

## Conclusions

There is a scope for more additions and inclusions in the national program for better care of CLHIV in India. Neurocognitive assessment is not a part of standard care of HIV-infected children in India. Neurocognitive lags in CLHIV negatively impact educative capabilities, social adaptations, and overall social integration. Authors recommend periodic neurocognitive assessments of CLHIV and moderation in their medical care and social care guided by such assessments. Early detection of cognitive deficits and timely modifications in socioenvironmental factors may improve the long-term outcomes for affected children.
